# Development of a highly sensitive immunohistochemical method to detect neurochemical molecules in formalin-fixed and paraffin-embedded tissues from autopsied human brains

**DOI:** 10.3389/fnana.2015.00022

**Published:** 2015-03-03

**Authors:** Satoshi Goto, Ryoma Morigaki, Shinya Okita, Shinji Nagahiro, Ryuji Kaji

**Affiliations:** ^1^Department of Motor Neuroscience and Neurotherapeutics, Institute of Health Biosciences, Graduate School of Medical Sciences, Tokushima UniversityTokushima, Japan; ^2^Parkinson’s Disease and Dystonia Research Center, Tokushima University Hospital, Tokushima UniversityTokushima, Japan; ^3^Department of Neurosurgery, Institute of Health Biosciences, Graduate School of Medical Sciences, Tokushima UniversityTokushima, Japan; ^4^Department of Clinical Neuroscience, Institute of Health Biosciences, Graduate School of Medical Sciences, Tokushima UniversityTokushima, Japan

**Keywords:** immunohistochemistry, polymer staining, tyramide signal amplification, avidin biotin complex, humans, brain, striatum, neuroanatomy

## Abstract

Immunohistochemistry (IHC) is a valuable method for identifying discrete neurochemical molecules by the interaction of target antigens with validated antibodies tagged with a visible label (e.g., peroxidase). We have developed an immunostaining method that is highly sensitive in detection of neurochemical antigens. Our IHC method, which we call the PBTA method, involves a hybrid protocol that implements aspects of both the polymer and avidin-biotin-complex (ABC) methods in combination with biotin-tyramide amplification. When using [Met]-enkephalin as a target antigen, the sensitivity of the PBTA method for IHC was more than 100-fold higher compared with the polymer and ABC methods. In addition, its sensitivity for enzyme-linked immunosorbent assay was about 1,000-fold higher compared with the ABC method. We examined the utility of our IHC method for both chromogenic and fluorescence detection systems used to visualize neurochemical peptides and proteins in formalin-fixed, paraffin-embedded tissues from autopsied human brains. The results convincingly demonstrate that under optimal conditions, our IHC method is highly sensitive without increasing non-specific background activities. Our IHC method could be a powerful tool for detection and visualization of neurochemical antigens that are present even in trace amounts in autopsied human brains.

## Introduction

In the fields of neuroanatomy and neuropathology, immuno­histochemistry (IHC) is a powerful tool for identifying discrete neurochemical molecules (e.g., neurotransmitters and their receptors) by the interaction of target antigens with specific antibodies tagged with a visible label (e.g., peroxidase). Because of their sensitivity in detecting antibodies applied to formalin-fixed paraffin-embedded (FFPE) preparations, both the avidin biotin complex (ABC; Hsu et al., 1981) and polymer (Sabattini et al., 1998) methods are most commonly used in neuroanatomical and neuropathological studies of human FFPE tissues. However, a long-standing criticism regarding the IHC method is the lack of sensitivity in detecting antigens present in tiny or scarcely detectable amounts in the FFPE tissues (for review see, van der Loos, 2007; Warford et al., 2014). Even with validated antibodies, it is often difficult to obtain specific and efficient immunoreaction products by the ABC or polymer staining technique in FFPE tissues, particularly those obtained from *autopsied* brains. This difficulty is frequently encountered, for example, in macroscopic images of immunostained human striatal sections prepared for visualizing striatal compartments composed of the striosomes and extrastriosomal matrix (Graybiel and Ragsdale, 1978). Increasing evidence suggests the relevance of the striosome and matrix domains to multiple human neurologic and neuropsychiatric disorders (for a review, see Crittenden and Graybiel, 2011). On the FFPE tissue from autopsied human brains, [Met]-enkephalin (MEnk) is one of the most reliable IHC markers to visualize striatal compartments with heightened density of MEnk labeling in the striosomes (Goto et al., 1990, 2013).

We have developed a highly sensitive IHC technique to localize neurochemical peptides and proteins in the human brain. The principal procedures involved in this sensitive technique are as follows. After incubation with primary antibodies, the brain sections were incubated with a **P**olymer staining reagent to introduce a large number of peroxidase molecules (Sabattini et al., 1998). The sections were then incubated with **B**iotin-**T**yramide signal amplification reagents to obtain the catalytic local deposition of a reporter (i.e., biotin) via the action of tyramide with peroxidase (Bobrow et al., 1989; Adams, 1992). This was followed by incubation with the **A**BC reagent to obtain a final visible label (i.e., peroxidase) (Hsu et al., 1981). For the purposes of this study, we named this IHC technique as the “**PBTA**” method. In this article, we describe the utility of the PBTA staining method in both chromogenic and fluorescence detection systems for visualizing MEnk and other neurochemical antigens in FFPE striatal tissues from autopsied human brains.

## Subjects and methods

### Autopsied human brain and tissue preparation

Human brains were obtained at autopsy from 10 neurologically normal individuals (mean age ± S.E.M., 59.5 ± 10.5). All procedures involving postmortem human brain tissue were approved by the Ethical Review Committee of the Tokushima University. Routinely, the brain tissue was fixed in 10% neutral formalin for about 3 weeks, and then embedded in paraffin. All the paraffin-embedded tissue blocks that we used here had been stored for more than 3 years at room temperature. In this study, 4-μm-thick brain sections were prepared on a microtome and mounted onto MAS-coated glass slides (Matsunami Glass, Osaka, Japan).

### IHC on human brain tissue

The sections were deparaffinized in xylene, immersed in decreasing concentrations of ethanol, and rehydrated in water. Endogenous peroxidase activity was blocked with 1% H_2_O_2_ in water for 5 min. All sections for immunostaining were processed for microwave-enhanced antigen retrieval. Slide-mounted sections immersed in 0.01 M sodium citrate buffer (pH 6.0) were placed for 15 min in a 700-W microwave oven at maximum power (Shi et al., 1991). After several rinses in phosphate-buffered saline (PBS, pH 7.2), endogenous avidin and biotin activities were blocked (Wood and Warnke, 1981) using the Avidin/Biotin Blocking Kit (Vector, Burlingame, CA, USA). Following several rinses in PBS, sections were further blocked in a PBS-BSA solution containing 3% bovine serum albumin (BSA) for 60 min at room temperature. They were then processed for the different immunostaining protocols described below, all of which were carried out at room temperature. No detergents (e.g., Triton-X100, Tween-20) were used in any of the IHC procedures.

### Polymer staining

The sections were incubated with rabbit polyclonal antibody against MEnk (AB5026 from Millipore, St. Louis, MO, USA; 1:100, 1:1,000, or 1:10,000) or without the anti-MEnk antibody for 18 h in PBS-BSA. After several rinses in PBS, they were incubated with the polymer staining reagent by using the Histofine Simple Stain Kit (Nichirei, Tokyo, Japan) for 30 min. After several rinses in PBS, the bound peroxidase was visualized by incubating the sections with a solution containing 0.05% 3,3′-diaminobenzidine (DAB; Merck, Darmstadt, Germany) and 0.01% H_2_O_2_ in 0.05 M Tris-HCl (pH 7.4) for 10 min. After several rinses in water, the immunostained sections were dehydrated and cover-slipped with Malinol (Muto Pure Chemicals, Tokyo, Japan).

### ABC staining

The sections were incubated with rabbit polyclonal antibody against MEnk (AB5026 from Millipore; 1:200, 1:2,000, or 1:20,000) or without the anti-MEnk antibody for 18 h in PBS-BSA. After several rinses in PBS, the sections were incubated with secondary biotinylated antibodies against rabbit IgG (Vector; 1:200) for 30 min. After several rinses in PBS, they were incubated with the Vectastain Elite ABC reagent for 30 min (Vectastain Elite ABC Kit; Vector). After several rinses in PBS, the bound peroxidase was visualized by incubating the sections with a solution containing 0.05% DAB (Merck) and 0.01% H_2_O_2_ in 0.05 M Tris-HCl (pH 7.4) for 10 min. After several rinses in water, the immunostained sections were dehydrated and cover-slipped with Malinol (Muto Pure Chemicals).

### PBTA-DAB staining

**Figure 1:**
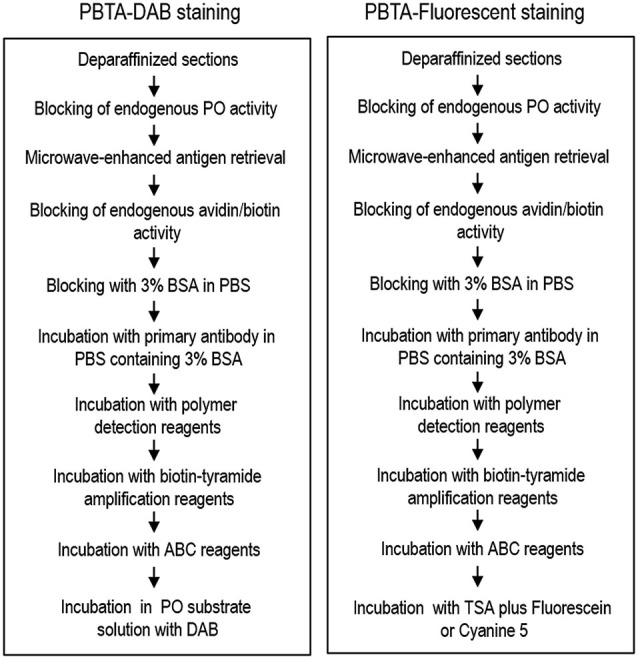
**Overview of the protocol for the PBTA method**. Protocols for the PBTA-DAB (*left*) and PBTA-Fluorescent (*right*) methods are shown. PO, peroxidase; BSA, bovine serum albumin; ABC, avidin biotin complex; DAB, diaminobenzidine.

The sections were incubated with rabbit polyclonal antibody against MEnk (AB5026 from Millipore; 1:50,000, 1:500,000, or 1:5,000,000) or without the anti-MEnk antibody for 18 h in PBS-BSA. After several rinses in PBS, the sections were incubated with the polymer staining reagent (Histofine Simple Stain Kit; Nichirei, Tokyo, Japan) for 30 min. After several rinses in PBS, they were processed for tyramide signal amplification (TSA) using the TSA Biotin System (Perkin Elmer, Boston, MA, USA). The sections were incubated in Biotinyl Tyramide (amplification reagent) Working Solution that was made by diluting Biotinyl Tyramide Stock Solution (Perkin Elmer) 1:50 using 1X Plus Amplification Diluent (Perkin Elmer, FP1135) for 30 min. After several rinses in PBS, the sections were incubated with the Vectastain Elite ABC reagent (Vector) for 30 min in PBS. After several rinses in PBS, the bound peroxidase was visualized by incubating the sections with a solution containing 0.05% DAB and 0.01% H_2_O_2_ in 0.05M Tris-HCl (pH 7.4) for 10 min. After several rinses in water, the immunostained sections were dehydrated and cover-slipped with Malinol (Muto Pure Chemicals). Overview protocol for the PBTA-DAB staining is shown in Figure 1 (*left*).

### PBTA-Fluorescent staining

For single antigen detection, the sections were incubated with rabbit polyclonal antibody against MEnk (AB5026 from Millipore, 1:20,000, 1:200,000, or 1:2,000,000) or mouse monoclonal antibody against choline acetyltransferase (ChAT; MAB9627 from Abnova, Taipei, Taiwan; 1:5,000) for 18 h in BSA-PBS. After several rinses in PBS, the sections were incubated with the polymer-staining reagent (Histofine Simple Stain Kit; Nichirei) for 30 min. After several rinses in PBS, they were processed for TSA using the TSA Biotin System (Perkin Elmer). Sections were then incubated in the biotinyl tyramide amplification reagent. Working solution was prepared by diluting Biotinyl Tyramide Stock Solution (Perkin Elmer) 1:50 using 1X Plus Amplification Diluent (Perkin Elmer) for 30 min. After several rinses in PBS, the sections were incubated with Vectastain Elite ABC reagent (Vector) for 30 min in PBS. After several rinses in PBS, the bound peroxidase was visualized by incubation with the TSA system with Cyanine3 or Fluorescein (Perkin Elmer) for 15 min. After several rinses in PBS, the sections were then cover-slipped with PBS containing 10% glycerol. An overview of the protocol for PBTA-Fluorescent staining is shown in Figure 1 (*right*).

For dual antigen detection, sections were first incubated in PBS-BSA containing rabbit polyclonal antibody against tyrosine hydroxylase (TH; 1:200,000) (Sato et al., 2008) or rat monoclonal antibody against the dopamine D1 receptor (D1R; D2944 from Sigma-Aldrich; 1:300,000) for 18 h. Bound antibodies were detected by the PBTA-Fluorescent staining protocol for single antigen detection with Cyanine3. To remove the bound antibodies, the stained sections were incubated in 0.1 M glycine-HCl (pH 2.2) for 30 min followed by incubation in PBS for 1 h. After blocking the peroxidase and avidin/biotin activities according to methods described above, the sections were incubated for 18 h in PBS-BSA containing rabbit polyclonal antibody against MEnk (AB5026 from Millipore; 1:200,000) or mouse monoclonal antibody against ChAT (MAB9627 from Abnova; 1:5,000). After several rinses in PBS, the bound antibodies were detected by the PBTA-Fluorescent staining protocol for single antigen detection with Fluorescein (Perkin Elmer). After several rinses in PBS, the sections were cover-slipped with PBS containing 10% glycerol.

### Digital images

Macroscopic images were captured by an Epson ES-2200 color image scanner (Seiko Epson Co., Suwa, Nagano, Japan) using the 24-bit color mode. Microscopic images were captured by an Olympus BX51 microscope (Olympus, Tokyo, Japan) equipped with a digital camera DP40 using the objective lens of Plan ApoN (1.25×/0.04), Plan ApoN (2×/0.08), UPlan FLN (4×/0.13), UPlan FLN (10×/0.30), UPlan FLN (20×/0.50), UPlan FLN (40×/0.75), or UPlan FLN (100×/1.30 oil). The digital images were imported into Adobe Photoshop CS4 and processed digitally for adjustments of contrast, brightness, and color balance.

### Enzyme-linked immunosorbent assay (ELISA) for MEnk

Stock solutions containing MEnk (1.0 mg/ml of free base; Sigma-Aldrich) were prepared in water and aliquots were stored at −20°C. Plates were washed 3 times between each step described below with the wash buffer (PBS containing 0.05% Tween 20). Stock MEnk solutions were diluted to 50 ng/ml in 0.1 M PB (pH 7.4), and covalently coupled to the wells (50 μl/well) of 96-well Iwaki ELISA plates (Asahi Glass Co., Tokyo, Japan) by overnight incubation at 37°C, according to a modification of the method reported by Denning et al. (2008). After adding 100 μl of PBS-BSA to each well, the plates were incubated for 18 h at 4°C. Thereafter, 50 μl of PBS-BSA containing anti-MEnk antibody at the indicated dilution was added to each well, and the plates were incubated for 18 h at room temperature. Wells containing 50 μl of PBS-BSA without anti-MEnk antibody served as background controls. Immunostaining was then carried out by the ABC or PBTA method in the manner described above. At each step, 50 μl of reagent solution was added to each well, and plate incubation was carried out for 30 min at room temperature. Color development was performed by adding 100 μl of 0.1 M citrate-phosphate buffer containing 0.2 mg/ml 3,3′,5,5′-tetramethylbenzidine (TMB) and 0.01% H_2_O_2_ (TMB Substrate Kit; Thermo Sci., Rockford, IL, USA). After incubating the plates for 5 min at room temperature, the absorbance at 652 nm was determined with a microplate reader (Bio-Rad Lab., Hercules, CA, USA).

### Statistical analysis

All experimental values are expressed as means ± S.E.M. Statistical significance was evaluated by the Mann-Whitney *U*-test. A *P* value of less than 0.05 was considered statistically significant.

## Results and discussion

### Comparison of IHC and ELISA detection of MEnk between ABC and PBTA methods

**Figure 2:**
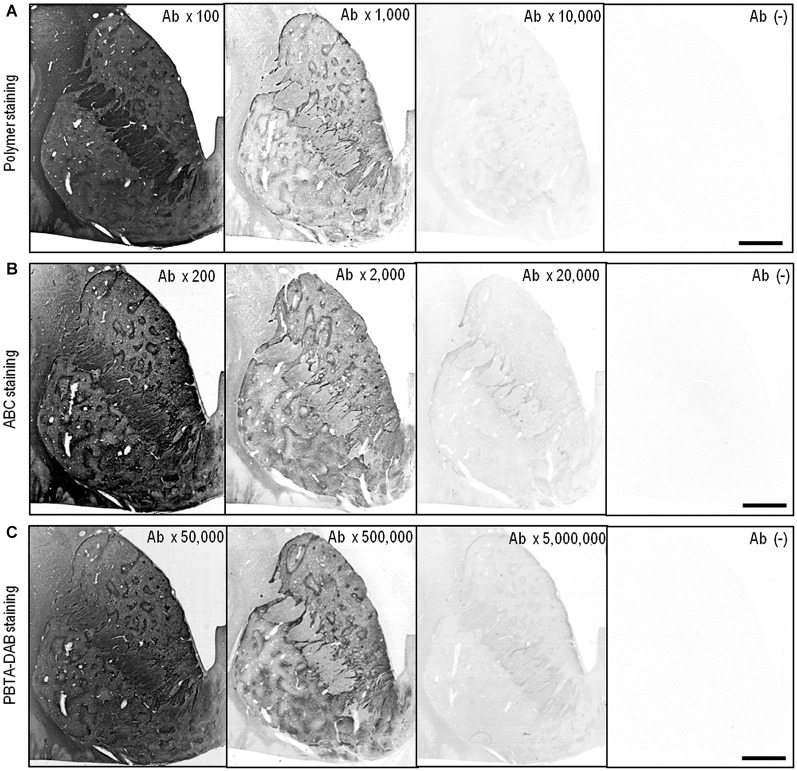
**IHC detection of MEnk by the polymer, ABC and PBTA methods**. Deparaffinized sections were processed for the IHC protocol of the polymer, ABC or PBTA-DAB method. **(A)** Frontal sections of the striatum stained by the polymer method with anti-MEnk antibody at the indicated dilutions of 1:100, 1:1,000, and 1:10,000. The section processed for the polymer staining protocol without anti-MEnk antibody [Ab (−)] is also shown. **(B)** Frontal sections of the striatum stained by the ABC method with anti-MEnk antibody at the indicated dilutions of 1:200, 1:2,000, and 1:20,000. The section processed for the ABC staining protocol without anti-MEnk antibody [Ab (−)] is also shown. **(C)** Frontal sections of the striatum stained by the PBTA-DAB method with anti-MEnk antibody at the indicated dilutions of 1:50,000, 1:500,000, and 1:5,000,000. The section processed for the PBTA-DAB staining protocol without anti-MEnk antibody [Ab (−)] is also shown. Note that optimal image of the striatal mosaic is obtained with the polymer method at the antibody dilution of 1:1,000, with the ABC method at the antibody dilution of 1:2,000, and with the PBTA-DAB method at the antibody dilution of 1:500,000. Scale bars: 4 mm.

Figure 2 illustrates the frontal sections of the striatum stained by the polymer (Figure 2A), ABC (Figure 2B) or PBTA-DAB (Figure 2C) method with anti-MEnk antibody at tenfold serial dilutions as indicated. Compartmentalized distribution of MEnk in the caudate nucleus and putamen was clearly visualized by the polymer method at the antibody dilution of 1:1,000, by the ABC method at the antibody dilution of 1:2,000, and by the PBTA-DAB method at the antibody dilution of 1:500,000. However, in all the polymer, ABC, and PBTA-DAB methods, a higher concentration of the antibody resulted in high non-specific background activities, while greater dilution of the antibody led to very weak staining results. Thus, the optimal dilution of anti-MEnk antibody for visualizing striatal mosaics with the PBTA-DAB method was more than 100-fold lower than those with the polymer and ABC methods.

**Figure 3:**
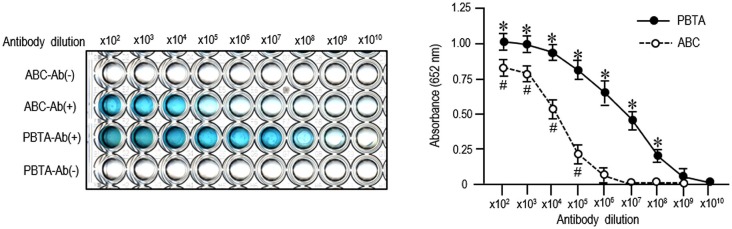
**ELISA detection of MEnk compared between ABC and PBTA methods**. MEnk peptides were covalently coupled to the wells of the ELISA plates (see the Methods). After blocking with PBS-BSA, PBS-BSA containing anti-MEnk antibody [Ab (+)] at the indicated tenfold dilution (from ×10^2^ to ×10^10^) was added to each well. Wells containing PBS-BSA without anti-MEnk antibody [Ab (−)] served as background controls. Immunostaining was carried out by the ABC or PBTA method. Color development was performed by using TMB a chromogen, and the absorbance at 652 nm was determined on a microplate reader. Each point is the mean ± S.E.M. (*n* = 4). ^#^*P* < 0.05, *P < 0.05 vs. the background controls at each dilution of antibody; Mann-Whitney *U*-test.

We employed an ELISA system for detecting MEnk by the ABC or PBTA-staining method using TMB as the chromogenic substrate. Results of both the ABC and PBTA methods (Figure 3) showed that the absorbance at 652 nm decreased in a dose-dependent manner, when the anti-MEnk antibody was added at the tenfold serial dilutions. Notably, specific reaction products could be detected up to an antibody dilution of 1:100,000 with the ABC method, and of 1:100,000,000 with the PBTA method. Thus, the detection limit in this ELISA system with the PBTA method appeared to be 1,000-fold higher in magnitude compared with that of the ABC method.

### PBTA-DAB staining locates MEnk in the human basal ganglia

**Figure 4:**
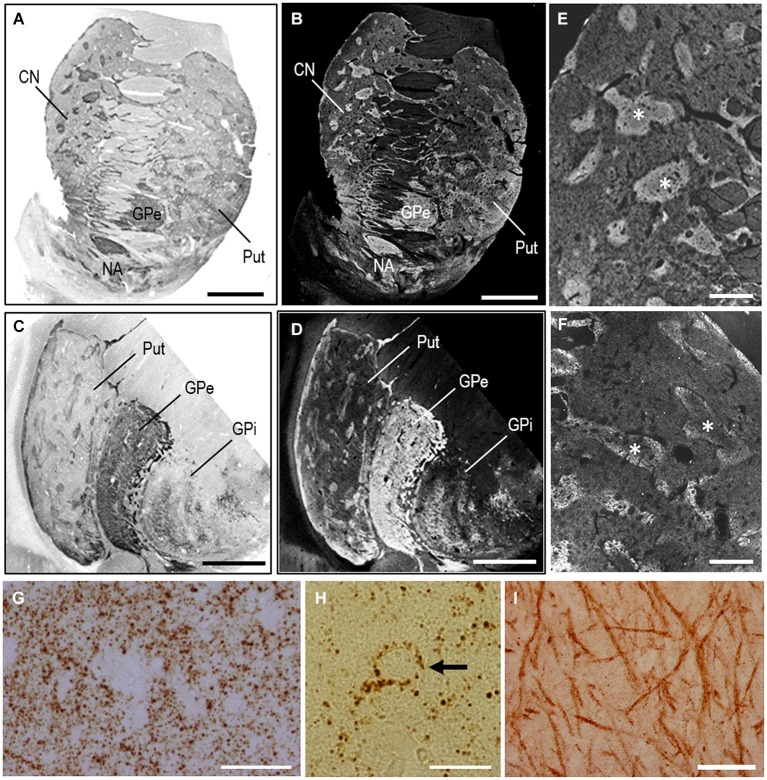
**MEnk localization in the basal ganglia as determined by PBTA-DAB staining**. Deparaffinized sections were processed for the PBTA-DAB staining protocol with anti-MEnk antibody at the dilution of 1:500,000. **(A,B)** A macroscopic image of the striatum **(A)** and its negative image **(B)**. **(C,D)** A macroscopic image of the lenticular nucleus **(C)** and its negative image **(D)**. **(E,F)** Low-power-magnified microscopic images of the caudate nucleus **(E)** and putamen **(F)**, where asterisks indicate striosomes marked by the MEnk staining. **(G)** High-power-magnified microscopic image of the striatal area, in which immunoreactive axon terminals are numerously found. **(H)** High-power-magnified microscopic image of an unstained cell surrounded by immunoreactive axon terminal. **(I)** High-power-magnified image of the GPe showing typical “wooly fibers”. CN, caudate nucleus; Put, putamen; GPe, globus pallidus externus; GPi, globus pallidus internus. Scale bars: **(A–D)** 4 mm; **(E)** 1 mm; **(F)** 500 μm; **(G)** 40 μm; **(H)** 20 μm; **(I)** 100 μm.

As an example of the applicability of PBTA-DAB staining on FFPE tissue from autopsied human brains, we show the staining results with anti-MEnk antibody in the basal ganglia (Figure 4). In frontal sections of the striatum (Figures 4A,B) and lenticular nucleus (Figures 4C,D), the striatal mosaic was clearly found in both the caudate nucleus and putamen. Low-power-magnified microscopic images also showed discrete striatal patches enriched in MEnk in the caudate nucleus (Figure 4E) and putamen (Figure 4F). High-power-magnified microscopic images showed numerous immunoreactive dots, presumably, reflecting axon terminals of the enkephalinergic cells in the striatum (Graybiel and Chesselet, 1984), where they often delineated the cell bodies of the unstained neurons (Figures 4G,H). In the globus pallidus externus (GPe), there were numerous immunoreactive axon terminals of the striatal projection neurons showing a typical “woolly fibers” pattern (Figure 4I). Thus, the MEnk-staining morphology obtained by the PBTA method was identical to that reported previously with the ABC method (Goto et al., 1990).

### Application of PBTA-Fluorescent staining on FFPE striatal sections

**Figure 5:**
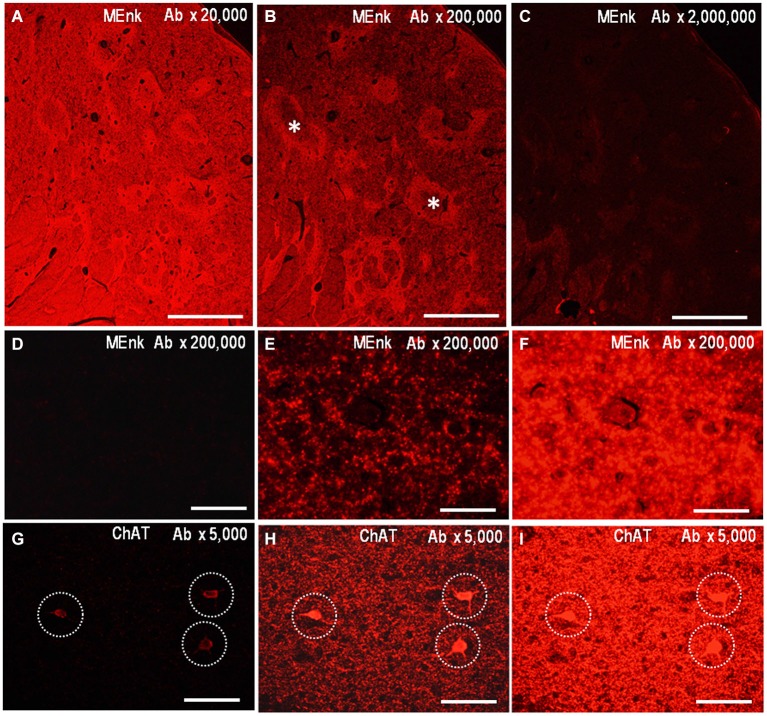
**Application of PBTA-Fluorescent staining to FFPE striatal sections**. Deparaffinized striatal sections were processed for the PBTA-Fluorescent (Cyanine 3) staining protocol with antibody against MEnk **(A–F)** or ChAT **(G–I)**.** (A–C)** Low-power-magnified images of the striatum stained with anti-MEnk antibody at a dilution of 1:20,000 **(A)**, 1:200,000 **(B)**, and 1:2,000,000 **(C)**. They were captured using a ×2 Plan ApoN objective lens with an exposure time (ET) of 20 ms. Asterisks indicate striosomes marked by the MEnk staining.** (D–F)** High-power-magnified images of a striatal area stained for MEnk at the antibody dilution of 1:200,000, when captured using a ×40 UPlan FLN objective lens with an ET of 0.2 ms **(D)**, 2.0 ms **(E)**, and 20 ms **(F)**. **(G–I)** High-power-magnified microscopic images of a striatal area stained for ChAT with the antibody dilution of 1:5,000, when captured using a ×20 UPlan FLN objective lens with an ET of 0.4 ms **(G)**, 4.0 ms **(H)**, and 20 ms **(I)**. Cell body staining is indicated by dashed open circles. Scale bars: **(A–C)** 1 mm; **(D–F)** 50 μm; **(G–I)** 100 μm.

The PBTA-Fluorescent method can also be applied to detect neurochemical molecules in FFPE tissue from autopsied human brains. We illustrate the low-power-magnified images of the striatum stained by the PBTA-Fluorescent (Cyanine 3) method with anti-MEnk antibody at a dilution of 1:20,000 (Figure 5A), 1:200,000 (Figure 5B), and 1:2,000,000 (Figure 5C), when captured using a ×2 Plan ApoN objective with an exposure time of 20 ms under an excitation and emission filter combination. Compartmentalized distribution of MEnk in the caudate nucleus was clearly visualized at the antibody dilution of 1:200,000; however, a higher antibody concentration resulted in increased non-specific background activities, while lower concentrations led to very weak staining results. Figures 5D–F show higher-power-magnified images of a striatal area stained for MEnk at the antibody dilution of 1:200,000, when captured using a ×40 UPlan FLN objective lens with an exposure time of 0.2 ms (Figure 5D), 2.0 ms (Figure 5E), and 20 ms (Figure 5F). A typical staining pattern with anti-MEnk antibody (Figure 4G) was found with a 2.0 ms exposure time. However this pattern was not visible with a 20-ms exposure time (optimal for visualizing the striatal mosaic in a low-power-magnified image captured with a x2 Plan ApoN objective lens) due to undesired intense fluorescent activities. Thus, higher signal intensities are required to obtain lower-power-magnified images for the representation of the striatal compartments. Notably, appropriate signal intensities also depend on what cellular components (e.g., cell bodies, axon terminals) are to be visualized at higher magnification. For example, we show the high-power-magnified microscopic images of a striatal area stained for ChAT with an optimal antibody dilution of 1:5,000, when captured using a ×20 UPlan FLN objective lens and an exposure time of 0.4 ms (Figure 5G), 4.0 ms (Figure 5H), and 20 ms (Figure 5I). The optimal exposure time of 4.0 ms (Figure 5H) yielded specific and strong fluorescent signals in both the cell bodies and surrounding fibers of cholinergic neurons. However, with the short exposure time of 0.4 ms (Figure 5G), only cell body staining could be detected and nerve fiber staining was absent. By contrast, with the long exposure time of 20 ms (Figure 5I), cell body staining was obscure due to the undesired intense activities of surrounding nerve fibers.

### PBTA-Fluorescent staining locates MEnk in the human basal ganglia

**Figure 6:**
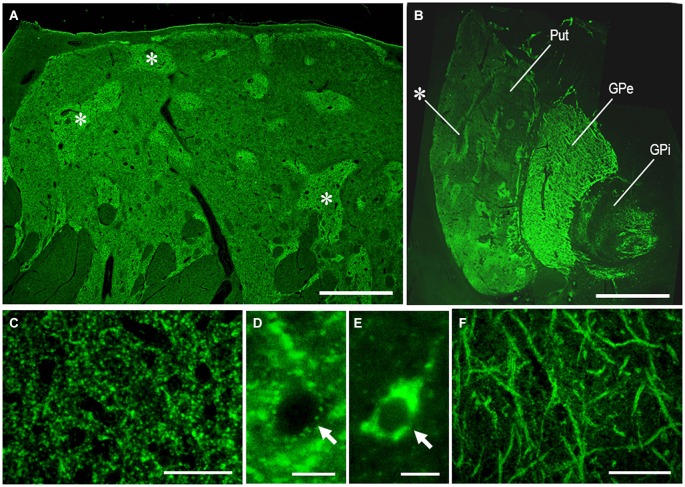
**MEnk localization in the basal ganglia as determined by PBTA-Fluorescent staining**. Deparaffinized sections were processed for the PBTA-Fluorescent (Fluorescein) staining protocol with anti-MEnk antibody at the dilution of 1:200,000. **(A)** Low-power-magnified microscopic image of the caudate nucleus, where the striatal mosaic is clearly found. Asterisks indicate examples of the striosomes marked by the MEnk staining. **(B)** A photomontage of the lenticular nucleus; asterisk indicates an example of the striosome in the putamen. **(C)** High-power-magnified microscopic image of the striatal area, in which immunoreactive axon terminals are numerously found. **(D)** High-power-magnified microscopic image of an unstained striatal cell surrounded by immunoreactive axon terminals. **(E)** High-power-magnified microscopic image of a stained striatal cell showing strong immunoreactivity within its soma. **(F)** High-power-magnified image of the GPe showing typical “wooly fibers”. Put, putamen; GPe, globus pallidus externus; GPi, globus pallidus internus. Scale bars: **(A)** 2.5 mm; **(B)** 4 mm; **(C)** 50 μm; **(D,E)** 10 μm; **(F)** 100 μm.

As an example of the applicability of PBTA-Fluorescent (Fluorescein) staining for FFPE tissue from autopsied human brains, we show the staining results with anti-MEnk antibody in the basal ganglia (Figure 6). In a low-power-magnified microscopic image of the caudate nucleus (Figure 6A) and in a photomontage of the lenticular nucleus (Figure 6B), the striatal mosaic was clearly seen in both the caudate nucleus and putamen. High-power-magnified microscopic images showed numerous immunoreactive dots in the striatum (Figure 6C), where they often delineated the cell bodies of unstained neurons (Figure 6D). Neurons possessing strong signals in their perikarya were occasionally found in the striatum (Figure 6E). In the GPe, there were numerous immunoreactive axon terminals of striatal projection neurons showing a typical “woolly fibers” pattern (Figure 6F). Thus, the MEnk-staining morphology obtained by the PBTA-Fluorescent method was identical to that with the PBTA-DAB method shown here.

### Dual antigen detection by the multiplexed PBTA-Fluorescent staining in striatal tissue

PBTA-Fluorescent staining can also be used for dual antigen detection in FFPE tissue from autopsied human brains. Brain sections were processed for PBTA-Fluorescent (Cyanine 3 or Fluorescein) staining with the first primary antibodies, and then for PBTA-Fluorescent (Fluorescein or Cyanine 3) staining with the second primary antibodies. Because the TSA-conjugated fluorophores remain deposited locally via covalent binding (Bobrow et al., 1989; Adams, 1992; Hunyady et al., 1996; Stack et al., 2014), the first primary antibodies can be removed at low pH (Okita et al., 2012; Koizumi et al., 2013) without any reduction of their specific fluorescent activity. The major advantage of the dual antigen detection system introduced here is that it allows use of first and second primary antibodies from the same species without species cross-reactivity. However, the residual peroxidase and avidin/biotin activities are required to be blocked before the sections are processed for PBTA-Fluorescent staining with the second primary antibodies.

**Figure 7:**
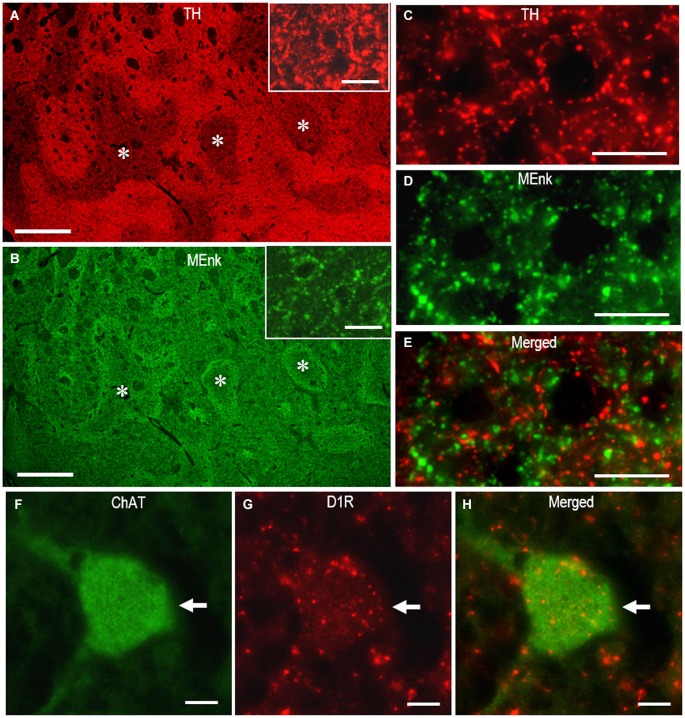
**Dual antigen detection by the multiplexed PBTA-Fluorescent staining of FFPE striatal sections**. Deparaffinized sections were processed for the double staining with the PBTA-Fluorescent protocols (see the Methods).** (A,B)** Low-power-magnified microscopic images of the striatum stained doubly with rabbit anti-TH **(A)** and anti-MEnk antibody **(B)**. The image insets in A and B show the striatal area stained for TH and MEnk, respectively, at a higher magnification. Note that TH labeling exhibits the striatal mosaic with a heightened density in the matrix compartment and is complementary to that of MEnk labeling. Asterisks indicate examples of the corresponding striosomes.** (C–E)** High-power-magnified microscopic images of the striatal area stained doubly with rabbit anti-TH **(C)**, and anti-MEnk antibody **(D)**. A merged image is also shown in **(E)**. Note that not all TH-immunoreactive dots co-localize with the MEnk-immunoreactive dots.** (F–H)** High-power-magnified microscopic images of a large-sized cell (arrow) stained doubly with mouse anti-ChAT **(F)** and rat anti-D1R antibody **(G)**. A merged image is also shown in **(H)**. Note that D1R-immunoreactive products are present in the cell body and proximal dendrites of the cholinergic cell. Scale bars: **(A,B)** 1 mm; **(C–E)** 20 μm; **(F–H)** 10 μm; (the image insets in **A** and **B**) 20 μm.

Figures 7A–E illustrate the microscopic images of striatal sections stained doubly for TH and MEnk with rabbit anti-TH and anti-MEnk antibody. At low-power magnification, TH immunoreactivity exhibited a striatal mosaic with heightened density in the matrix compartment (Figure 7A); this was complementary to that of MEnk labeling (Figure 7B). This was consistent with the previous finding with the ABC method on frozen sections of the human striatum (Holt et al., 1997). At high-power magnification (Figures 7C–E), TH-positive dots, largely of the axonal fibers originating from the midbrain dopaminergic cells (Howes et al., 2013), were distributed in a differential manner to MEnk-positive dots. The high-power-magnified images of striatal cells stained doubly for ChAT and D1R with mouse anti-ChAT and rat anti-D1R antibody are shown in Figures 7F–H. They clearly demonstrate that a cholinergic cell possesses the D1R-immunoreactive products in its cell body and proximal dendrites, consistent with a previous report (Khan et al., 2000). These observations indicate that irrespective of the host species of the antibodies used, the PBTA-Fluorescent method can be an efficient tool for double immunostaining.

## Conclusions

The PBTA method is a highly sensitive IHC system for detection of neurochemical molecules in FFPE tissue from autopsied human brains. Our results convincingly demonstrate that with optimal staining and imaging conditions, the PBTA method is highly efficient and does not increase non-specific background activities. This allows macroscopic visualization of the compartmental organization of target antigens in the striatum. When using MEnk as a target antigen, the sensitivity of the PBTA method for IHC was more than 100-fold higher compared with the polymer and ABC methods. Moreover, the sensitivity of the PBTA-method for ELISA was about 1,000-fold higher compared with the ABC method. Although not shown here, detection sensitivity of the PBTA-DAB method can be further increased by addition of an enhancement to the final peroxidase/DAB reaction, using imidazole (Straus, 1982), metals (Hsu and Soban, 1982), or silver-gold intensification (Rossi et al., 1991; Goto et al., 1992). The PBTA method can be applied to frozen (or vibratome) sections from either human or experimental animal brains; however, use of this method is usually limited to cases when the antibodies have very low specific activity. We also demonstrated the applicability of the PBTA method for fluorescence detection of neurochemical antigens. Repeated PBTA protocols with the same species’ primary antibodies can be used for dual antigen detection without species cross-reactivity. Based on these observations, we suggest that the PBTA method could be a powerful tool for detection and visualization of neurochemical antigens that are present only in trace amounts in FFPE tissue from autopsied human brains.

## Conflict of interest statement

The authors declare that the research was conducted in the absence of any commercial or financial relationships that could be construed as a potential conflict of interest.

## References

[B1] Adams J. C. (1992). Biotin amplification of biotin and horseradish peroxidase signals in histochemical stains. J. Histochem. Cytochem. 40, 1457–1463. 10.1177/40.10.1527370 1527370

[B2] Bobrow M. N. Harris T. D. Shaughnessy K. J. Litt G. J. (1989). Catalyzed reporter deposition, a novel method of signal amplification. Application to immunoassays. J. Immunol. Methods 125, 279–285. 10.1016/0022-1759(89)90104-x 2558138

[B3] Crittenden J. R. Graybiel A. M. (2011). Basal ganglia disorders associated with imbalances in the striosome and matrix compartments. Front. Neuroanat. 5:59. 10.3389/fnana.2011.00059 21941467 PMC3171104

[B4] Denning G. M. Ackermann L. W. Barna T. J. Armstrong J. G. Stoll L. L. Weintraub N. L.. (2008). Proenkephalin expression and enkephalin release are widely observed in non-neuronal tissues. Peptides 29, 83–92. 10.1016/j.peptides.2007.11.004 18082911

[B5] Goto S. Hirano A. Matsumoto S. (1990). Met-enkephalin immunoreactivity in the basal ganglia in Parkinson’s disease and striatonigral degeneration. Neurology 40, 1051–1056. 10.1212/wnl.40.7.1051 2192300

[B6] Goto S. Kawarai T. Morigaki R. Okita S. Koizumi H. Nagahiro S.. (2013). Defects in the striatal neuropeptide Y system in X-linked dystonia-parkinsonism. Brain 136(Pt. 5), 1555–1567. 10.1093/brain/awt084 23599389

[B7] Goto S. Nagahiro S. Ushio Y. Hofer W. (1992). A simple enhancement method for the silver-gold-intensified diaminobenzidine reaction in the light microscopic immunoperoxidase technique. J. Histochem. Cytochem. 40, 1423–1425. 10.1177/40.9.1506678 1506678

[B8] Graybiel A. M. Chesselet M. F. (1984). Compartmental distribution of striatal cell bodies expressing [Met] encephalin-like immunoreactivity. Proc. Natl. Acad. Sci. U S A 81, 7980–7984. 10.1073/pnas.81.24.7980 6440146 PMC392277

[B9] Graybiel A. M. Ragsdale C. W. (1978). Histochemically distinct compartments in the striatum of human, monkeys and cat demonstrated by acetylthiocholinesterase staining. Proc. Natl. Acad. Sci. U S A 75, 5723–5726. 10.1073/pnas.75.11.5723 103101 PMC393041

[B10] Holt D. J. Graybiel A. M. Saper C. B. (1997). Neurochemical architecture of the human striatum. J. Comp. Neurol. 384, 1–25. 10.1002/(sici)1096-9861(19970721)384:1<1::aid-cne1>3.0.co;2-5 9214537

[B11] Howes O. D. Williams M. Ibrahim K. Leung G. Egerton A. McGuire P. K.. (2013). Midbrain dopamine function in schizophrenia and depression: a post-mortem and positron emission tomographic imaging study. Brain 136, 3242–3251. 10.1093/brain/awt264 24097339 PMC3808688

[B12] Hsu S. M. Raine L. Fanger H. (1981). Use of avidin-biotin-peroxidase complex (ABC) in immunoperoxidase techniques: a comparison between ABC and unlabeled antibody (PAP) procedures. J. Histochem. Cytochem. 29, 577–580. 10.1177/29.4.6166661 6166661

[B13] Hsu S. M. Soban E. (1982). Color modification of diaminobenzidine (DAB) precipitation by metallic ions and its application for double immunohistochemistry. J. Histochem. Cytochem. 30, 1079–1082. 10.1177/30.10.6182185 6182185

[B14] Hunyady B. Krempels K. Harta G. Mezey E. (1996). Immunohistochemical signal amplification by catalyzed reporter deposition and its application in double immunostaining. J. Histochem. Cytochem. 44, 1353–1362. 10.1177/44.12.8985127 8985127

[B15] Khan Z. U. Gutiérrez A. Martín R. Peñafiel A. Rivera A. de la Calle A. (2000). Dopamine D5 receptors of rat and human brain. Neuroscience 100, 689–699. 10.1016/s0306-4522(00)00274-8 11036203

[B16] Koizumi H. Morigaki R. Okita S. Nagahiro S. Kaji R. Nakagawa M.. (2013). Response of striosomal opioid signaling to dopamine depletion in 6-hydroxydopamine-lesioned rat model of Parkinson’s disease: a potential compensatory role. Front. Cell. Neurosci. 7:74. 10.3389/fncel.2013.00074 23730270 PMC3656348

[B17] Okita S. Morigaki R. Koizumi H. Kaji R. Nagahiro S. Goto S. (2012). Cell type-specific localization of optineurin in the striatal neurons of mice: implications for neuronal vulnerability in Huntington’s disease. Neuroscience 202, 363–370. 10.1016/j.neuroscience.2011.11.059 22155493

[B18] Rossi F. van der Want J. J. Wiklund L. Strata P. (1991). Reinnervation of cerebellar Purkinje cells by climbing fibres surviving a subtotal lesion of the inferior olive in the adult rat. II. Synaptic organization on reinnervated Purkinje cells. J. Comp. Neurol. 308, 536–554. 10.1002/cne.903080404 1865016

[B19] Sabattini E. Bisgaard K. Ascani S. Poggi S. Piccioli M. Ceccarelli C.. (1998). The EnVision++ system: a new immunohistochemical method for diagnostics and research. Critical comparison with the APAAP, ChemMate, CSA, LABC and SABC techniques. J. Clin. Pathol. 51, 506–511. 10.1136/jcp.51.7.506 9797726 PMC500802

[B20] Sato K. Sumi-Ichinose C. Kaji R. Ikemoto K. Nomura T. Nagatsu I.. (2008). Differential involvement of striosome and matrix dopamine systems in a transgenic model of dopa-responsive dystonia. Proc. Natl. Acad. Sci. U S A 105, 12551–12556. 10.1073/pnas.0806065105 18713855 PMC2527949

[B21] Shi S. R. Key M. E. Kalra K. L. (1991). Antigen retrieval in formalin-fixed, paraffin-embedded tissues: an enhancement method for immunohistochemical staining based on microwave oven heating of tissue sections. J. Histochem. Cytochem. 39, 741–748. 10.1177/39.6.1709656 1709656

[B22] Stack E. C. Wang C. Roman K. A. Hoyt C. C. (2014). Multiplexed immunohistochemistry, imaging and quantitation: a review, with an assessment of Tyramide signal amplification, multispectral imaging and multiplex analysis. Methods 70, 46–58 10.1016/j.ymeth.2014.08.016 25242720

[B23] Straus W. (1982). Imidazole increases the sensitivity of the cytochemical reaction for peroxidase with diaminobenzidine at a neutral pH. J. Histochem. Cytochem. 30, 491–493. 10.1177/30.5.6176617 6176617

[B24] van der Loos C. M. (2007). A focus on fixation. Biotech. Histochem. 82, 141–154. 10.1080/10520290701375302 17852085

[B25] Warford A. Akbar H. Riberio D. (2014). Antigen retrieval, blocking, detection and visualization systems in immunohistochemistry: a review and practical evaluation of tyramide and rolling circle amplification systems. Methods 70, 28–33. 10.1016/j.ymeth.2014.03.001 24631890

[B26] Wood G. S. Warnke R. (1981). Suppression of endogenous avidin-binding activity in tissues and its relevance to biotin-avidin detection systems. J. Histochem. Cytochem. 29, 1196–1204. 10.1177/29.10.7028859 7028859

